# Transvenous Lead Extraction: Do We Need to Explore Alternative Techniques?

**DOI:** 10.19102/icrm.2019.100204

**Published:** 2019-02-15

**Authors:** Christopher R. Ellis

**Affiliations:** ^1^Vanderbilt Heart and Vascular Institute, Nashville, TN, USA

**Keywords:** Lead extraction, lead management, minimally invasive surgery, transvenous lead extraction

At this time, an ongoing dilemma regarding transvenous lead extraction (TLE) remains. How do we determine using a noninvasive assessment whom to send directly for open surgical removal? As has been observed across many high-volume extraction centers, the case complexity, total dwell time, and indications for TLE have changed. Frequently, we are now referred cases in elderly pacemaker-dependent patients with more than four leads in place, including epicardial, abdominal, and previously fractured or aborted extraction cases. The consequence of these lead management disasters now bubbling to the surface is that their addressment will require innovative thinking by both electrophysiologists and cardiac surgeons alike.

Questions that we now encounter frequently include the following: should we extract old or nonfunctional epicardial leads when a transvenous system is infected? When should we opt to extract from a right internal jugular approach? What if a short fragment of lead with exposed wire is left after partial extraction and abandoned? In this issue of *The Journal of Innovations in Cardiac Rhythm Management*, a series of cases from Miami managed by way of nontraditional approaches utilizing minimally invasive surgical techniques is presented.^1^ Of note, one essential relationship that must be in place for a successful extraction program is collaboration between cardiac surgery and cardiac electrophysiology. There must be an open dialogue about how to manage increasing case complexity, with a lead extraction team put into place to help facilitate successful outcomes, both before a case is attempted and in instances when failure occurs, necessitating sternotomy.

The flowchart in Azarrafiy and Carrillo’s **Figure 1** provides a scheme for minimally invasive surgical consideration. However, each decision point is still debatable due to a lack of prospective data. In the era of vacuum-aspiration catheters and improved snare techniques, one question to consider is when is a vegetation really too large to attempt management via a transvenous approach? Also, how can we assess how compressible or soft a large mass is by echocardiography, transesophageal echocardiography, or intracardiac echocardiography? How old is too old for a lead to be effectively removed? When is it reasonable to attempt minimally invasive surgical extraction in a patient with a previous coronary bypass grafting (ie, in a non-“virgin” chest)? We have overall found the removal of epicardial leads via direct minithoracotomy to be fairly straightforward at our institution; however, even with right lateral atriotomy, partially extracted or fractured lead fragments can be quite challenging to locate when attempting a minimally invasive extraction. In some cases, we have reversed course and opted to instead remove lead remnants successfully via a transvenous right internal jugular approach following failed open surgical removal.

A competing balance exists in the shortcomings of either approach, which really should promote a greater collaborative effort. The tools for TLE are quite effective cutting tools that are designed to hug the lead, vaporize fibrotic material, or slice through calcified adhesions and, in some cases, are more effective than a surgeon with a scalpel and scissors in removal. Alternatively, having the ability to quickly place a hemostatic plug and stitch over a hole in the superior vena cava (SVC), right atrium (RA), or right ventricle (RV) and place epicardial pacing hardware or replace a tricuspid valve can only be accomplished by a skilled cardiac surgeon. A notable case was recently referred to Vanderbilt University Medical Center. The patient was 84 years old with prior coronary bypass and was pacemaker-dependent with class IV congestive heart failure and moderate–severe aortic stenosis with a 4-cm enterococcal vegetation. TEE showed severe tricuspid regurgitation with a combined lead age of 88 years. The endocardial RV implantable cardioverter-defibrillator (ICD) lead and unipolar coronary sinus pacing lead were removed by TLE and the RA and RV pacing leads from 1997 were freed to the SVC. Leads were encased in a large vegetation (which could not be vacuum-aspirated). Next, a minithoracotomy approach was performed to remove the mass, remaining leads, and epicardial left ventricular (LV) leads (failed with a threshold of > 5 V for both). New epicardial RA and LV leads were implanted and a tissue tricuspid valve replacement was performed **(Figure 1)**.

The presented Miami experience from Azarrafiy and Carrillo is quite helpful as evidence that cases can be managed without midline sternotomy and still yield high success and excellent survival to hospital discharge rates. As we enter an era of leadless pacing, leadless defibrillation, and a surge in LV assist device use, the complexity of extraction nightmares that are expected to continue to surface will undoubtedly continue to rise. Thinking outside the box may be the only way to ensure that our patients don’t end up in the “box.”

## Figures and Tables

**Figure 1:**
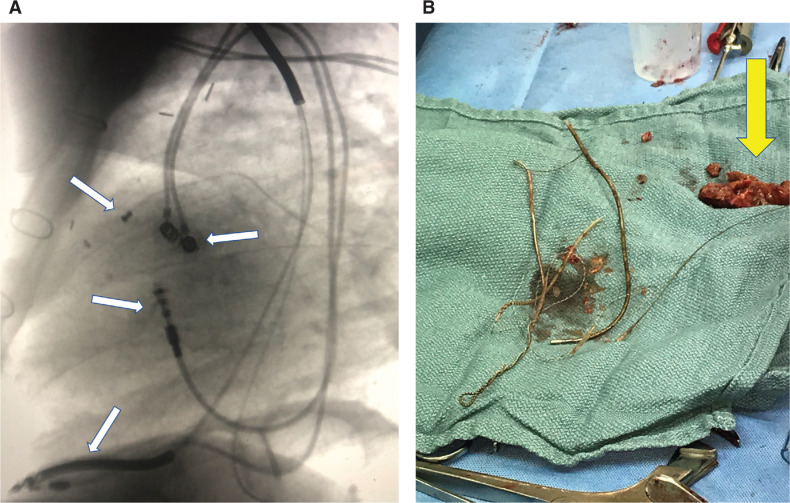
Complex extraction case in a very high-risk patient performed by way of hybrid TLE with minithoracotomy. **A:** Image shows 1388 RA and RV leads (Abbott Laboratories, Chicago, IL, USA), a 6947 RV ICD lead (Medtronic, Minneapolis, MN, USA), two epicardial LV leads, and a 4193 coronary sinus lead (Medtronic, Minneapolis, MN, USA) (white arrows). A CardioMEMS™ device (Abbott Laboratories, Chicago, USA) was also previously in place in the pulmonary artery branch. The patient underwent prior bypass surgery via midline sternotomy. **B:** The RA and RV lead remnants were removed and new epicardial RA and LV leads were implanted. Tissue tricuspid valve replacement was performed. A 4.9-cm × 3-cm mass (*Enterococcus* sp.) was removed (yellow arrow). Original figure courtesy of Christopher Ellis.
